# Clinical Outcomes Following Dual-Frequency Noninvasive Monopolar Radiofrequency Treatment for Facial Laxity and Lower Face Lifting: A Prospective Multicenter Study

**DOI:** 10.7759/cureus.104546

**Published:** 2026-03-02

**Authors:** Robert A Weiss, Jordan Wang, Barry DiBernardo, Ashish C Bhatia

**Affiliations:** 1 Dermatology, Maryland Laser, Skin, and Vein Institute, Baltimore, USA; 2 Dermatology, Laser and Skin - Dermatology, Devon, USA; 3 Plastic Surgery, New Jersey Plastic Surgery, Montclair, USA; 4 Dermatology, Oak Dermatology, Naperville, USA

**Keywords:** adverse events, dual-frequency radiofrequency, facial laxity, global aesthetic improvement scale, lower face lifting, monopolar radiofrequency, noninvasive monopolar radiofrequency, noninvasive skin tightening, patient satisfaction score, prospective multicenter study

## Abstract

Introduction

Facial aging is a common dermatological and plastic surgical concern. As patients today wish to avoid surgical intervention, noninvasive monopolar radiofrequency (NMRF) can be a useful tool to address this concern, but results have varied by target depth. A dual-frequency NMRF system (6.78 MHz and 2 MHz) is designed to support depth-dependent tissue heating across dermal and subdermal planes. This study aimed to evaluate short-term clinical outcomes and safety in a prospective multicenter trial.

Methods

Thirty-nine subjects at four clinical sites completed the study. The protocol allowed up to three treatments spaced four weeks apart; however, all subjects completed the study after two sessions. Treatments were delivered at three different depths (shallow, middle, and deep), as indicated by the target condition and facial anatomical zone. Standardized clinical photography was taken at baseline, 30- and 90-day timepoints. A five-point Global Aesthetic Improvement Scale (GAIS) was assessed by the principal investigators at the 30- and 90-day assessments, and patients subjectively scored their satisfaction using a study-specific patient satisfaction score (PSS; six-point scale). GAIS results were evaluated for statistical significance; satisfaction was summarized descriptively with exact 95% CIs. Any adverse events were documented.

Results

A total of 39 subjects received two treatment sessions. Clinician-scored GAIS achieved three or above by 84.6% (33/39; 95% CI, 69.5-94.1) of subjects at 30-day and 92.3% (36/39; 95% CI, 79.1-98.4) of subjects at 90-day timepoints (p=0.0293 and 0.0008, respectively). Patient satisfaction was also high, scoring of four or above for 79.5% of participants at 30-day (31/39; 95% CI, 64.5-89.2%) and 84.6% at 90-day (33/39; 95% CI, 70.3-92.8%). There were no long-lasting adverse events.

Conclusion

Dual-frequency NMRF, applied with depth-dependent settings in this study, was associated with significant short-term improvement and high satisfaction at 30 and 90 days, with no persistent adverse events. These findings support further evaluation of this noninvasive RF approach for facial aging.

## Introduction

Facial aging is a universal concern, and improvement of age-related changes requires stimulation of mechanisms to restore lost collagen and elastin [1,2]. Multi-level etiology is comprised of loss of volume, namely: deep bone resorption occurs with thinning of the periosteum and decreased blood supply, and mid-depth loss of volume is seen in the deeper fat layer. Contour changes arise from diminished elasticity and reduced support of the muscle and superficial musculoaponeurotic system (SMAS), resulting in the descent of the superficial fat pads. The basement membrane becomes compromised; senescence of fibroblasts leads to poorly organized collagen fibers with interstitial spaces in the dermis and loss of elasticity of the elastic fibers. Finally, surface changes are seen in thinning and flattening of the epidermis, accompanied by a patchy stratum granulosum and a poorly organized stratum corneum [1-3].

Clinically, facial aging is characterized by the appearance of wrinkles, accentuation of nasolabial folds and marionette lines, brow ptosis, undereye hollowing, lower facial soft-tissue ptosis along the mandibular border, and submental laxity [4]. Successful treatment of skin aging should address all layers of the skin and subcutaneous tissues [3]. Topical regimens tend to address only superficial targets. Other current approaches such as ablative lasers may be invasive and associated with extensive patient downtime [5]. Patients often avoid these more invasive modalities due to pain, prolonged downtime, and procedure-related risks, factors that also drive demand for non-surgical alternatives. Current patient expectations have led to a trend toward noninvasive treatments with controlled pain and downtime, such as noninvasive monopolar radiofrequency (NMRF), which received FDA clearance in 2002 [6]. Several generations of NMRF have been developed with many reports in the literature showing efficacy [7-9]. Pain, however, has remained a fairly constant complaint.

NMRF uses a handpiece with an active (delivery) electrode placed on the target skin and a large-area return electrode (grounding pad) attached elsewhere on the body [10]. In bipolar radiofrequency (RF) systems, both the delivery and return electrodes are incorporated within the handpiece [10]. In NMRF, RF current travels through the skin and underlying soft tissues (including the collagen-rich dermis) to a distant return electrode. As the current encounters tissue impedance (resistive load), electrical energy is converted into heat (Joule heating) [11]. Because a localized region of elevated temperature (“hot spot”) develops where the delivery electrode contacts the skin, some form of cooling is required to protect the epidermis from damage. Commonly available NMRF systems operate at a single frequency, typically 6.78 MHz, and deeper penetration is limited by exposure time, causing significant pain and protracted downtime. Unwanted adverse events, such as burning and blistering, have been common [12].

A dual-frequency NMRF system used in the present study delivers standard 6.78 MHz for superficial-to-intermediate dermal heating and can be combined with 2.0 MHz to support deeper subdermal heating. Depth-dependent heating is supported by frequency-dependent tissue electrical properties and preclinical modeling; however, tissue-level confirmation in humans (including effects on specific deep structures such as the SMAS) was not assessed in this clinical study. The purpose of this prospective, Institutional Review Board-approved multi-site study was to evaluate short-term clinical outcomes and safety of dual-frequency NMRF treatment for facial laxity and lower face lifting.

## Materials and methods

**Figure 1:**
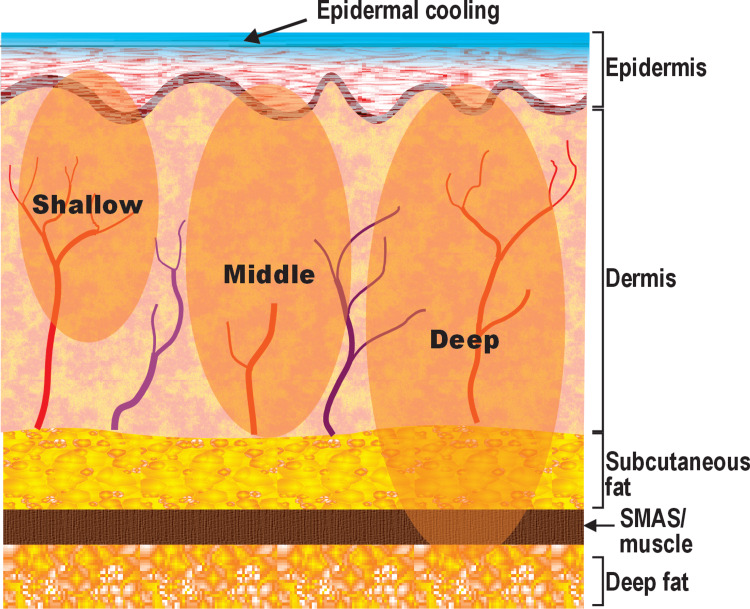
Approximate target depths and tissue zones for the shallow, middle, and deep settings of the dual-frequency noninvasive monopolar radiofrequency (NMRF) device SMAS: superficial musculoaponeurotic system. Image credit: Original internal illustration created by Cynosure Lutronic Inc. (Goyang, South Korea; data on file) using CorelDRAW (Corel Corporation, Ottawa, Canada). Reproduced with permission.

A total of 39 subjects (37 female patients, two male patients) were enrolled over four geographically distinct sites with Fitzpatrick skin types I-IV, classified according to the Fitzpatrick skin phototype system [13] (type I, five; type II, 17; type III, 13; type IV, four) and ages from 28-70 years (average 54.4 years). Subjects received up to three treatment sessions spaced four weeks apart at shallow, middle, or deep mode (Figure 1), as appropriate for the target condition and facial anatomical zone; however, all subjects received two sessions in this study.

The treatment protocol was uniform for the four centers, and prospective subjects were enrolled according to the inclusion and exclusion criteria as follows.

Inclusion criteria included being male or female participants, 22 to 70 years of age, and willing to undergo treatment with the study device; willing to maintain their current diet and exercise routine throughout the duration of the study; understanding and accepting the study conditions; and able to provide written informed consent.

Exclusion criteria included (if it was a female participant) pregnancy, either currently or within the three months prior to the study, lactating, or planning pregnancy during the study period; having an implanted pacemaker or similar electrical device; having metal implants that could interfere with transmission of energy to the target tissue; having permanent fillers or facial implants; infected tissue in the treatment area; having received previous surgical or cosmetic procedures in the treatment area in the last three months that could interfere with the novel device procedure (including but not limited to dermabrasion, chemical peels, laser skin resurfacing, fat augmentations, topical retinoids, and radiofrequency treatments); history of smoking within the month prior to the study; having unrealistic treatment expectations.

The dual-frequency NMRF device (XERF, Cynosure Lutronic Inc., Goyang, South Korea) is operated at both 6.78 MHz and 2.0 MHz frequencies. The handpiece tip delivers RF energy to the tissue homogeneously across the entire tip, without hotspots at the edges (“spider-pattern”). Adjustable cryogen gas cooling (levels one to three) provides surface cooling to help protect the superficial epidermis from overheating. Tip surface temperature is displayed with each shot to provide real-time monitoring, and RF output is automatically terminated if the tip surface temperature exceeds 43°C. Skin cooling also aids pain prevention. Pulse energy is delivered using a “wave fit pulse” RF pulse train composed of a variable number of subpulses with individually adjusted energy and timing according to the selected depth and energy level settings.

The study was performed in accordance with Good Clinical Practice (GCP), as required by EN ISO 14155, the Declaration of Helsinki, Investigational Device Exemption (21 CFR Part 812), Protection of Human Subjects (45 CFR Part 46), and other applicable FDA regulations; regulations of other relevant regulatory authorities; and conditions imposed by the reviewing IRB. The study was approved as a nonsignificant risk study by the Allendale Institutional Review Board, Old Lyme, CT, USA (Protocol no. LMP24001). All patients gave their written informed consent to participate in the study and for the scientific use of their photographic data. The study device and research support were provided by the manufacturer (Cynosure Lutronic Inc.), which also supported data management and/or statistical programming. The investigators were responsible for clinical conduct, interpretation of results, and the final content of the manuscript.

The treatment protocol included facial cleansing and application of a thin film of ultrasound gel as a sliding agent to facilitate smooth movement and help distribute cooling evenly for comfort and safety. No topical anesthesia was used. The largest handpiece tip, EFFECTOR 60, was used for all patients, and sliding mode, stamping mode, or a combination was used depending on the target site. Sliding mode was used for broad, less contoured areas to provide uniform coverage with better comfort, whereas stamping mode was used for contoured or bony areas for stable contact and localized energy delivery; a combined approach was used when both coverage and focal tightening were desired. The number of shots delivered to each site and the total energy delivered to all treatment areas (in joules) were recorded.

Assessments

Clinical photographs were obtained using a digital SLR camera at baseline and at 30 and 90 days after the final treatment. Sites followed a standardized photography approach (consistent patient positioning and facial expression, fixed views, and a consistent background) to the extent feasible in routine clinical practice. Despite standardization efforts, minor differences in illumination and color balance between visits may occur and can influence visual interpretation. No image enhancement was performed other than cropping and de-identification. Site principal investigators performed Global Aesthetic Improvement Scale (GAIS) scoring using clinical photography at each time point [14]. GAIS was assessed by the treating investigators and was not blinded; independent blinded photographic review was not performed. A score of three or above was considered effective. Patients assessed their satisfaction (patient satisfaction score, PSS) using a six-point scale, with one representing extreme dissatisfaction and six representing significant satisfaction. A score of four or above was used to determine positive satisfaction. Pain was also scored on an 11-point numerical rating scale (NRS; 0=no pain, 10=worst possible pain) [15]. GAIS responder rates (three or above) at each follow-up time point were evaluated post hoc against a 70% reference proportion using a one-sided exact binomial test in R (ver 4.5.1, R Foundation for Statistical Computing, Vienna, Austria) [16]. Subject satisfaction was summarized descriptively as responder rate (four or above) with exact 95% confidence intervals; no formal hypothesis testing was performed for this secondary endpoint. GAIS and NRS are nonproprietary instruments and do not require a license for use in clinical research reporting; the PSS was developed for this study and is not a licensed instrument. This post hoc benchmark analysis was exploratory and intended to contextualize responder rates; no adjustment for confounders or repeated-measures modeling was performed.

## Results

The final per-protocol population comprised 39 subjects, all of whom successfully completed all treatment and follow-up requirements. These data were included in the statistical analysis. Energy levels ranged from one to 10, depending on the state of the skin, degree of severity of the condition being treated, and the treatment site, then titrated to patient tolerability (procedural discomfort) to achieve an acceptable level of pain while maintaining effective treatment delivery. Overall, the total shot count delivered per treatment (sum across all treated facial and neck zones, both sides) ranged from 300 to 677, with a mean of 568.8. The total energy delivered to all treated sites ranged from 28,738.9 J to 92,996.3 J, with an average of 56,391.2 J (56.4 kJ), corresponding to an average of 100.7 J per shot. Pain levels were generally mild to moderate (average score, 4.2) without anesthesia.

Clinician-scored GAIS results at 30 and 90 days were very good and statistically significant. At 30 days, 33/39 (84.6%) subjects had GAIS scores of three or above (p=0.0293). At 90 days, 36/39 (92.3%) subjects had GAIS scores of three or above (p=0.0008). Subject satisfaction was also high, with responder rates (PSS of four or above) of 79.5% (31/39; 95% CI, 64.5%-89.2%) at 30 days and 84.6% (33/39; 95% CI, 70.3%-92.8%) at 90 days. In general, results at the 30-day assessment were maintained and slightly improved at the 90-day assessment. The GAIS outcomes are summarized in Table 1. 

**Table 1:** Clinician-scored global aesthetic improvement scale (GAIS) at 30 and 90 days after the final treatment ^†^P values were calculated using a one-sided exact binomial test against a 70% reference proportion; responders were defined as GAIS ≥3. GAIS or Global Aesthetic Improvement Scale (1=worse, 2=no change, 3=improved, 4=much improved, 5=very much improved) [14]; 95% CIs are exact binomial confidence intervals; SD, standard deviation.

Assessment	Time point (days)	Responders n/N (%, CI)	GAIS score, mean score ± SD	P value^†^
GAIS (scored from 1-5)	30	33/39 (84.6%; 95% CI 69.5-94.1)	3.26 ± 0.75	0.0293 (one-sided exact binomial test vs p_0_=0.70; x=33, n=39)
	90	36/39 (92.3%; 95% CI 79.1-98.4)	3.46 ± 0.68	0.0008 (one-sided exact binomial test vs p_0_=0.70; x=36, n=39)

There were no long-lasting adverse events other than expected mild and transient sequelae such as erythema and edema. In cases where these findings persisted longer, all resolved without treatment.

Clinical photography

**Figure 2:**
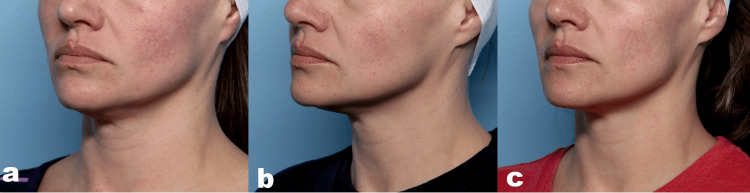
Clinical photographs of a 42-year-old female patient (a) At baseline, (b) 30 days, (c) and 90 days after the final treatment. Visible improvement in skin laxity and redness is observed at 30 days and is maintained with further improvement at 90 days. Treatment settings are described in the text.

**Figure 3:**
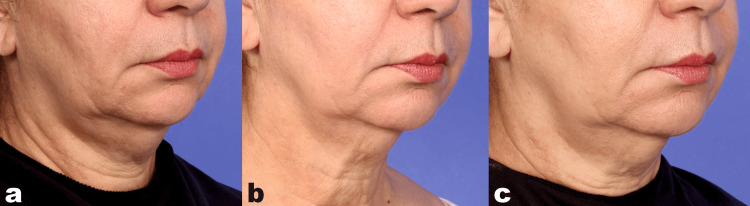
Clinical photographs of a 60-year-old female patient (a) At baseline, (b) 30 days, (c) and 90 days after the final treatment. Overall improvement of the skin is apparent, with lifting of the lower face and tightening at the jawline, with lifting and smoothing of neck wrinkles; these findings continued to improve at 90 days. Treatment settings are described in the text.

Representative results are shown in Figures 2, 3.

Figure 2 demonstrates a 42-year-old female patient seeking improvement in redness, skin texture, and laxity. She received two treatment sessions with the same approach in both sessions. The forehead was treated with the middle depth setting at levels four to six. The midface was treated with the middle setting at level four. The lower face was treated with two depth settings, namely middle followed by deep settings at level four. The submental and neck areas were treated with the middle depth setting at levels three to four. At the 30-day assessment (Figure 2b), there were visible improvements in both laxity and redness; these results were maintained and improved at the 90-day assessment (Figure 2c).

Figure 3 shows a 60-year-old female patient, with overall tightening apparent at 30 days (Figure 3b), including lower face lifting and smoothing of neck rhytids. Further improvement is seen at the 90-day assessment (Figure 3c). She received two treatment sessions. In both treatments, the deep setting was selected as the treatment depth at levels five to seven.

## Discussion

NMRF has been available, typically at 6.78 MHz, from the early 2000s onwards [7-9,17]. Despite iterative refinements such as epidermal cooling, conventional continuous wave NMRF has remained limited by procedural discomfort and variability in clinical response across patients and anatomical regions [7-9].

A dual-frequency NMRF device was investigated to address limitations of conventional continuous wave-based systems by using an RF pulse train (“wave fit pulse”) composed of a variable number of subpulses with individually adjusted energy and timing, rather than a fixed subpulse sequence typical of traditional NMRF. This concept is corroborated by Hwang et al.’s work, which evaluated pain using this novel pulse structure as well as dual frequencies [18]. In the present study, the mean in-treatment pain was 4.2/10 without anesthesia. In prior monopolar RF facial laxity studies using Thermage platforms (Solta Medical, Bothell, WA, USA), mean during-procedure pain scores of approximately six to seven out of 10 have been reported (6.06 for Thermage CPT and 6.9 for Thermage FLX, the latter despite ketorolac pre-treatment) [8,9]. Although cross-study comparisons are inherently limited by differences in design and treatment parameters, these findings suggest favorable tolerability in our cohort. We hypothesize that Wave Fit Pulse (Cynosure Lutronic Inc., Goyang, South Korea), by fractionating energy delivery with optimized timing and integrated cooling, may reduce peak heating and superficial thermal accumulation, thereby attenuating cutaneous nociceptor activation.

The ideal temperature range generated by the electrothermal reaction in the target dermal tissue should be between 45°C and 65°C to achieve protein denaturation and heat shock protein production. Mild coagulation with immediate shrinkage is anticipated at higher delivered energy settings. Stimulation of the wound healing process, comprising collagenesis and elastinogenesis followed by tissue remodeling, is the goal [19]. At the lower temperatures, the heat-labile hydrogen bonds holding tropocollagen together denature, allowing the fibers to separate and subsequently renature on cooling, stimulating collagen reorganization [20]. In addition, heat shock proteins (HSPs), especially HSP47, enhance the wound healing process by acting as molecular chaperones to assist in proper protein folding during the production of new collagen from active fibroblasts [21].

Traditional single-frequency NMRF systems may be less effective at reliably inducing remodeling beyond the mid-reticular dermis. The dual-frequency NMRF system evaluated here is designed to support deeper subdermal heating using the 2.0 MHz mode; however, the depth of tissue effect was not directly measured in this human cohort, and mechanistic interpretation should be considered inferential. Lower-frequency monopolar RF is expected to penetrate more deeply (approximately proportional to 1/√f) due to frequency-dependent tissue electrical properties, and modeling data show that 2 MHz produces broader and deeper thermal effects with greater heating in the upper subcutaneous fat than 6.78 MHz [22].

Park et al., based out of South Korea, performed a pre-clinical in vivo trial with this system in mini pigs [23], and Hong et al. performed a histological study using the same porcine model [24]. Histologic findings immediately after treatment were consistent with structural changes in treated tissue [24]. The earlier in vivo mini pig histological study also reported collagenesis and findings suggestive of subdermal remodeling in the porcine model [23].

A separate experiment performed in the in vivo animal porcine model assessed core dermal temperatures in vivo using the same NMRF system at low (level one), nominal (level five), and high (level 10) settings, at the shallow, middle, and deep settings for all four tips. Dermal temperatures peaked at approximately 50-70°C across energy settings, while skin surface temperatures remained below 43°C under monitored conditions, supporting controlled heating with epidermal protection [23]. These findings suggest potential translatability to clinical practice; however, replication in independent cohorts and controlled studies is warranted. The magnitude of clinical improvement and tolerability observed at 90 days appears broadly consistent with outcomes reported for contemporary monopolar RF facial rejuvenation protocols [7-9,17]. However, cross-study comparisons are inherently limited by differences in patient populations, endpoints, anesthesia use, and treatment parameters. Future randomized split-face studies comparing this dual-frequency wave fit pulse protocol with a standard single-frequency 6.78 MHz regimen (e.g., Thermage FLX), using standardized photography and blinded assessment, would provide more definitive comparative evidence.

This study has limitations. Although it was prospective, it lacked a control or comparative arm, which limits causal inference and direct comparison with established NMRF protocols. The study was unblinded, and GAIS assessments were performed by the treating principal investigators; therefore, evaluator bias cannot be excluded. Despite a standardized photography protocol, minor visit-to-visit differences in lighting and color balance may have affected qualitative visual comparisons; future studies should use fully standardized imaging with independent, blinded photographic assessment. Follow-up was limited to 90 days after the final treatment; longer-term assessment is warranted given that extracellular matrix remodeling may continue for several months after NMRF. In addition, tissue-level depth of effect was not directly assessed in humans, and inferences regarding deeper structural targeting should be interpreted with caution. Preclinical porcine histology provides biological plausibility for subdermal remodeling but does not confirm specific deep anatomic effects in this clinical cohort [23]. Only two male participants were included, which limits generalizability to male patients. The study population comprised Fitzpatrick skin types I-IV; therefore, generalizability to individuals with Fitzpatrick skin types V-VI is uncertain. Finally, the sample size limited statistical power and precluded meaningful subgroup analyses (e.g., by sex, age, baseline severity, or Fitzpatrick skin type).

## Conclusions

In this prospective multicenter study, dual-frequency NMRF using depth-dependent settings was associated with short-term improvement in the clinical appearance of facial wrinkles and laxity, high patient satisfaction, and no persistent adverse events through 90 days after the final treatment. Given the study design limitations, these findings should be interpreted as preliminary and hypothesis-generating rather than definitive evidence of mechanism or depth of effect. Future randomized comparator studies incorporating blinded independent photographic assessment, objective outcome measures, and longer follow-up are warranted to confirm durability and further characterize clinical response.
